# Coronary sinus lactate at reperfusion: A predictor of atrial fibrillation after coronary artery bypass grafting

**DOI:** 10.1016/j.xjon.2026.101971

**Published:** 2026-07-24

**Authors:** Arjun Verma, Borami Shin, Sameer Hirji, Michael Gilfeather, Tommaso Hinna Danesi, Ashraf A. Sabe, Thoralf M. Sundt, Sary F. Aranki

**Affiliations:** Division of Cardiac Surgery, Mass General Brigham, Harvard Medical School, Boston, Mass

**Keywords:** coronary artery bypass grafting, postoperative atrial fibrillation, coronary sinus sampling, atrial fibrillation risk model

## Abstract

**Objective:**

Postoperative atrial fibrillation (POAF) is the most common complication after coronary artery bypass grafting (CABG). This study explored the association of coronary sinus (CS) lactate levels with POAF after CABG.

**Methods:**

This was a single-center prospective cohort study of patients without a history of atrial fibrillation undergoing isolated CABG (December 2024 to February 2026). CS lactate was sampled before initiation of cardiopulmonary bypass (pre-CPB), before cross-clamp removal (pre-XC), and after cross-clamp removal (post-XC). POAF was defined as electrocardiogram-documented atrial fibrillation treated with amiodarone during the index hospitalization. Restricted cubic splines were used to define a binary cutoff of post-XC CS lactate at >3 mmol/L. Multivariable logistic regression was performed to assess the association between CS lactate elevation and POAF.

**Results:**

Of the 94 patients included, POAF occurred in 24 patients (26%). Patients with POAF were older and more commonly male but had similar median CPB time and XC duration. Patients with a post-XC lactate >3 mmol/L (n = 13/94, 14%) had significantly greater rates of POAF compared with those without lactate elevation (62% vs 20%, *P* = .001). After risk adjustment, post-XC lactate >3 mmol/L was associated with a 11.7-fold increase in odds of POAF (*P* = .010). Increasing age was also linked to greater likelihood of POAF. There was no significant association of POAF with CPB time, XC duration, pre-CPB CS lactate, or pre-XC CS lactate.

**Conclusions:**

Elevated CS lactate after cardiac reperfusion was associated with increased likelihood of POAF. Our findings suggest that CS lactate may be used to guide POAF prophylaxis.

**Figure:**
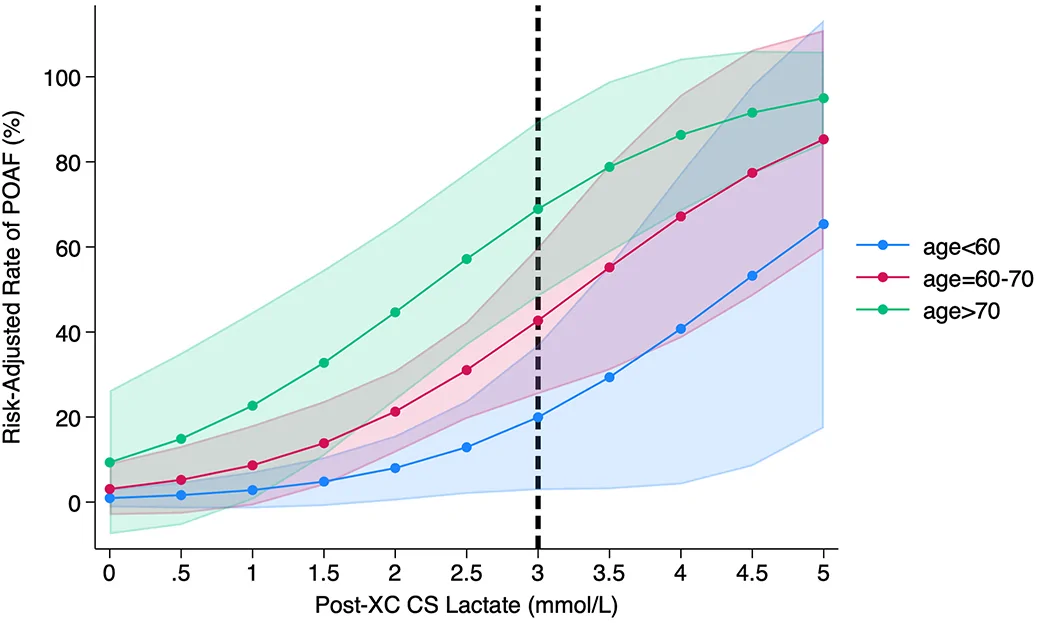
Risk of POAF increases with post-XC CS lactate, across strata of patient age.

**Central Message**

High coronary sinus lactate concentrations after cardiac reperfusion were associated with greater odds of postoperative atrial fibrillation after CABG.

**Perspective**

Postoperative atrial fibrillation is the most common complication after CABG, yet accurate prognostication remains elusive. We found that coronary sinus lactate >3 mmol/L after cardiac reperfusion was associated with a 11.7-fold increased risk of postoperative atrial fibrillation. Our findings suggest that coronary sinus lactate sampling can be used to inform deployment of targeted prophylaxis for postoperative atrial fibrillation.

Postoperative atrial fibrillation (POAF) is the most common complication after coronary artery bypass grafting (CABG), with a reported incidence of 17% to 40% in contemporary series.^1^^,^^2^ Although often transient, POAF is associated with increased resource use and long-term morbidity.^3^^,^^4^ Previous work has identified several risk factors for POAF, including advanced age, male sex, and preexisting left ventricular dysfunction.^5^^,^^6^ However, predictive models incorporating these characteristics (apart from older age) display poor performance upon external validation.^7^^,^^8^

The pathogenesis of POAF is multifactorial, incompletely understood, and thought to be driven by systemic and local metabolic stresses of cardiopulmonary bypass (CPB) superimposed on vulnerable atrial substrate.^9^ Lactate is a recognized marker of tissue hypoperfusion and metabolic distress, and it often is linked to adverse outcomes. For instance, Naik and colleagues^10^ evaluated 370 patients undergoing cardiac surgery and found that systemic hyperlactatemia (>4 mmol/L) was associated with a 3.5-fold increase in POAF. Models that incorporate measures of intraoperative myocardial injury (ischemia and reperfusion) may yield enhanced prognostication of POAF. As such, coronary sinus (CS) lactate sampling may better reflect cardiometabolic stress without being confounded by the degree of systemic illness; however, the relationship between CS lactate levels and POAF remains unexplored.

We leveraged a prospectively maintained, single-center database of CS lactate concentrations originally developed to examine the relationship between myocardial metabolic stress and postoperative outcomes after CABG. In the present analysis, we specifically evaluated the association between CS lactate levels and the development of POAF. Patient- and operative-level factors associated with elevated CS lactate concentrations were also secondarily assessed.

## Methods

### Study Design and Cohort

This was a single-center cohort study of patients undergoing CABG between December 2024 and February 2026 at the Mass General Brigham: Brigham and Women's Hospital. Data were prospectively collected as part of an institutional initiative designed to identify peri- and intraoperative risk factors for adverse outcomes after CABG. The present analysis focused on POAF, and therefore, those patients with a history of atrial fibrillation (AF) were excluded. Moreover, those requiring concomitant valve intervention and those who did not undergo aortic cross-clamping (XC) due to the presence of severe aortic atherosclerosis were removed (Figure 1). Finally, patients who received del Nido cardioplegia (n = 6) were excluded to preserve cohort homogeneity. This work was conducted under a protocol approved by the Mass General Brigham Institutional Review Board (no. 2018P001524, approved July 18, 2018). because of the low risk for patients, the need for patient consent was waived.

**Figure 1:**
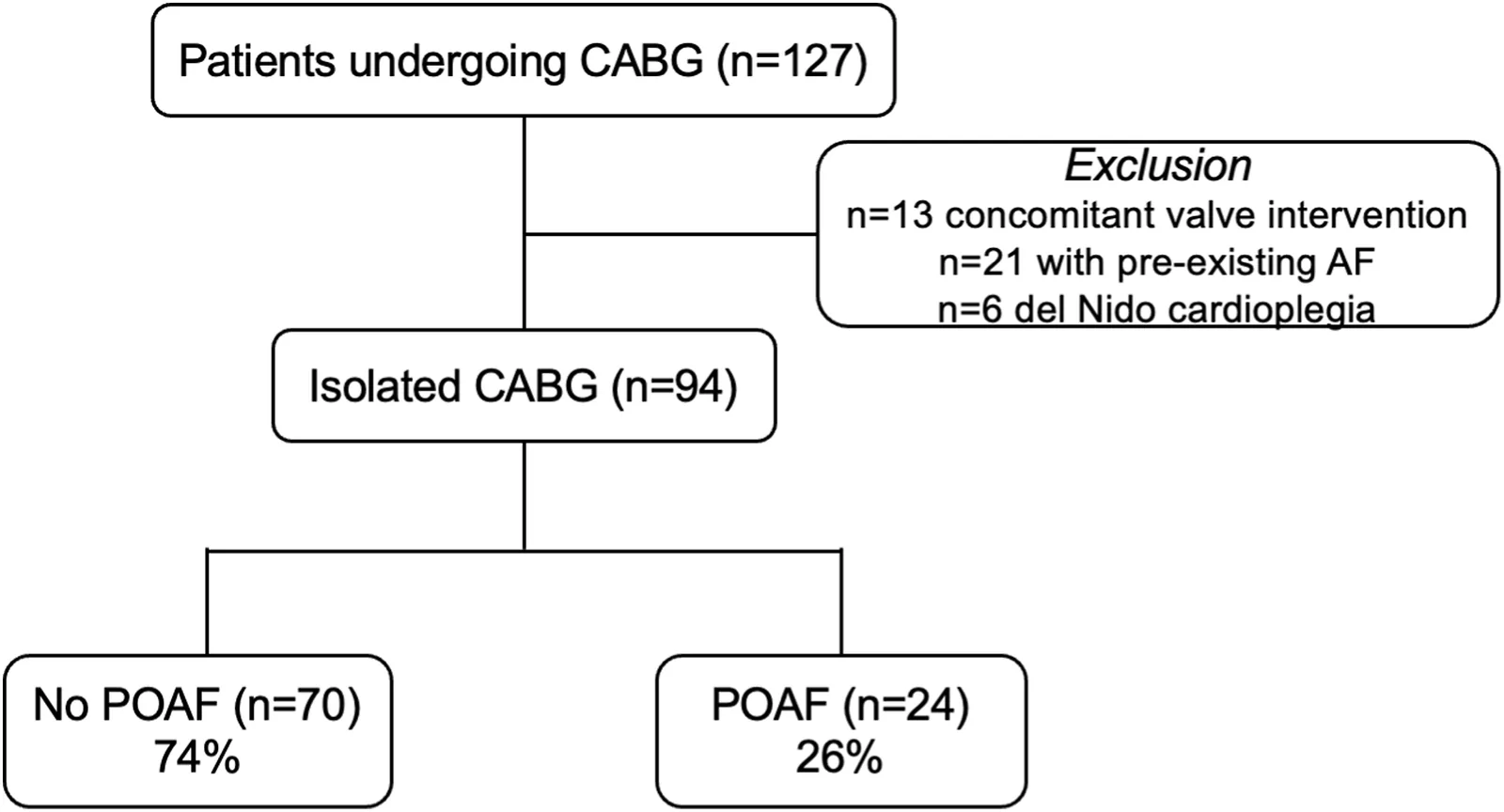
Cohort diagram. *CABG*, Coronary artery bypass grafting; *AF*, atrial fibrillation; *POAF*, postoperative atrial fibrillation.

### Surgical Approach

All operations were performed by 2 surgeons (S.F.A., B.S.). After the completion of traditional median sternotomy, the aorta and right atrium were cannulated, and CPB was initiated. Patients are allowed to passively cool (typically to 34 °C). After aortic XC, cardioplegic arrest was induced with antegrade and retrograde 8:1 whole-blood cardioplegia, with additional doses administered at 20-minute intervals. CS blood samples were collected via retrograde cannulation at 3 intraoperative time points. First, a sample was obtained before the initiation of CPB (pre-CPB) to represent a baseline. Second, in anticipation of aortic XC removal (5 minutes prior), an additional sample was taken (pre-XC). To obtain this sample, the left internal mammary artery was briefly opened to provide enough volume to the coronary circulation to be used for sampling. Lastly, another sample was obtained 5 minutes after aortic XC removal (post-XC). This time point reflects the status of the reperfused myocardium. Right atrial (RA) samples were also obtained at the pre-CPB and post-XC time points. Samples were analyzed immediately after collection using the RapidPoint 500e Blood Gas System (Siemens Healthineers). Patients are preoperatively maintained on beta-blockers for POAF prophylaxis. Postoperatively, beta-blockade is initiated as soon as clinically feasible. No other prophylactic strategies for AF were used, including amiodarone or left posterior pericardiotomy.

### Variable Definition

Age at operation, sex, and individual comorbidities (body mass index [BMI], type 2 diabetes mellitus, hyperlipidemia, hypertension, chronic lung disease, and chronic kidney disease stage 3 or greater) were among several baseline characteristics collected through review of the electronic medical record. Preoperative left ventricular ejection fraction and left ventricular mass (indexed to body surface area) were also recorded. Operative notes and perfusion records were reviewed to tabulate the number of bypassed vessels, performance of coronary endarterectomy, total CPB time, and aortic XC duration.

The primary outcome was POAF, defined as electrocardiographically documented AF requiring treatment with amiodarone during the index hospitalization. Amiodarone was administered if AF was noted to last >1 hour or if symptomatic for any duration. Continuous telemetry monitoring is performed for all patients until discharge. Secondary outcomes included in-hospital mortality and length of stay.

### Statistical Analysis

Continuous variables are reported as medians with interquartile ranges (IQR) and compared using the Mann-Whitney *U* test. Categorical variables are expressed as counts with percentages, with differences evaluated using the Pearson χ^2^ test. The strength and statistical significance of correlations were assessed with the Pearson correlation coefficient. Restricted cubic spline regression was used to assess the nonlinear relationship between CS lactate levels and the incidence of POAF (Figure 2). A cutoff value of >3 mmol/L was derived from the inflection point of the spline curve for post-XC CS lactate values. Subsequently, multivariable logistic regression was performed to evaluate the risk-adjusted association of elevated post-XC CS lactate with POAF. Covariates were selected using a combination of elastic net regularization with forced inclusion of clinically relevant variables. The final model included adjustment for age at operation, CPB duration, aortic XC duration, and number of distal anastomoses, as well as pre-CPB and pre-XC CS lactate concentrations. There was low concern for collinearity between independent variables, as the variance inflation factor was calculated to be less than 5 for all covariates. Model discrimination was assessed using the C-statistic, with 95% CIs generated using bootstrap validation with 200 resamples. Regression results are reported as adjusted odds ratios (aORs) with 95% CIs. All statistical analyses were performed using Stata, version 19.1 (StataCorp).

**Figure 2:**
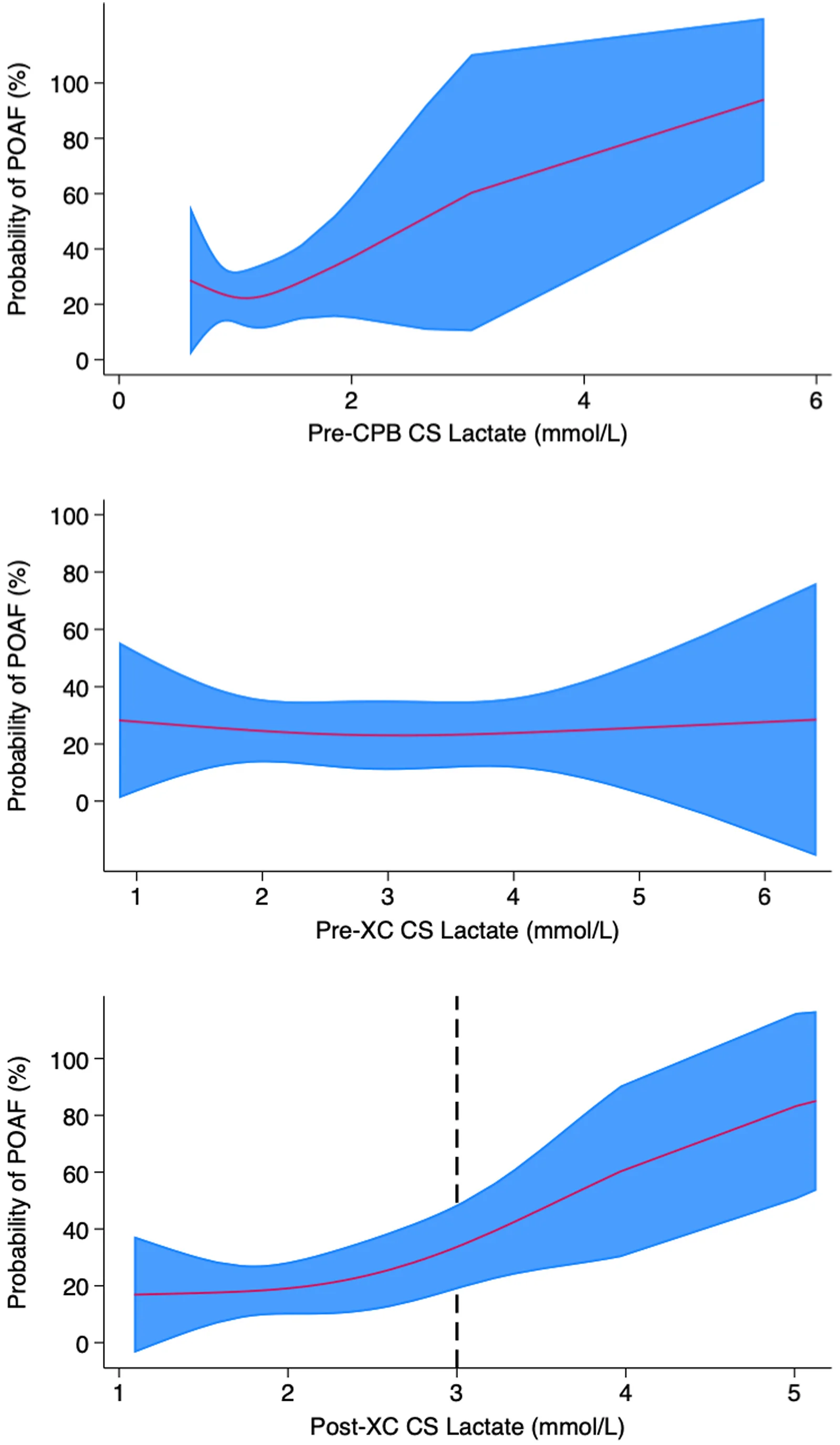
Restricted cubic spline analysis of coronary sinus (*CS*) lactate concentrations and the likelihood of postoperative atrial fibrillation (*POAF*). 95% CIs indicated by the *shaded blue regions*. *CPB*, Cardiopulmonary bypass; *XC*, crossclamp.

## Results

### Study Cohort

Of 94 patients meeting study criteria, 24 (26%) developed POAF. An additional 11 patients had AF documented on telemetry that did not meet criteria for amiodarone treatment and therefore were not included in the primary end point. Patients with POAF were older (median 72 [IQR, 67-77] vs 68 [IQR, 60-71] years, *P* = .010) and more commonly male (100 vs 77%, *P* = .010). Rates of hyperlipidemia, hypertension, type 2 diabetes mellitus, chronic lung disease, and chronic kidney disease were similar between those who did and did not develop POAF (Table 1). The distributions of BMI, preoperative left ventricular ejection fraction, and indexed left ventricular mass were also similar. Of note, patients with POAF had comparable CPB time (117 minutes [IQR, 92-165] vs 113 [IQR, 98-138] minutes, *P* = .51) and aortic XC durations (92 minutes [IQR, 73-112] vs 93 [IQR, 77-111] minutes, *P* = .73) with those who did not develop POAF. The distribution of the number of distal anastomoses did not differ between groups. Fifteen patients (16%) required endarterectomy of the left anterior descending coronary artery, with no difference in POAF rates (endarterectomy: 20% vs no endarterectomy: 27%, *P* = .59). Length of stay was significantly greater for those who developed POAF, compared with others (7 [IQR, 6-10] days vs 6 [IQR, 5-7] days, *P* = .006). There was 1 in-hospital mortality of a patient who developed POAF.

**Table 1:** Cohort characteristics stratified by development of POAF

Parameter	No POAF (n = 70)	POAF (n = 24)	*P*
Age, y	67.7 [59.5-71.1]	71.6 [66.8-77.0]	.011
Female sex	16 (22.9%)	0 (0.0%)	.010
BMI, kg/m^2^	28 [25-31]	29.5 [26.5-33.5]	.50
Preoperative LVEF, %	60 [55-65]	60 [58-65]	.23
Preoperative LV mass index, g/m^2^	88.6 [72.8-102.4]	77.3 [74.8-99.7]	.45
Hyperlipidemia	65 (92.9%)	22 (91.7%)	.85
Hypertension	59 (84.3%)	20 (83.3%)	.91
Diabetes	32 (45.7%)	7 (29.2%)	.16
Chronic lung disease	7 (10.0%)	3 (12.5%)	.73
Chronic kidney disease	11 (15.7%)	4 (16.7%)	.91
Number of bypasses			.57
2	9 (12.9%)	2 (8.3%)	
3	38 (54.3%)	17 (70.8%)	
4	19 (27.1%)	4 (16.7%)	
5	4 (5.7%)	1 (4.2%)	
Cardiopulmonary bypass time, min	113 [98-138]	116.5 [92-165]	.51
Aortic crossclamp time, min	93 [77-111]	91.5 [72.5-111.5]	.73
Endarterectomy	12 (17.1%)	3 (12.5%)	.59

*POAF*, Postoperative atrial fibrillation; *BMI*, body mass index; *LVEF*, left ventricular ejection fraction; *LV*, left ventricular.

### CS Lactate

Median CS lactate levels at pre-CPB, pre-XC, and post-XC were 1.0 [IQR, 0.9-1.3], 2.8 [IQR, 2.0-3.7], and 2.1 [IQR, 1.7-2.6] mmol/L, respectively (Figure E1). At baseline, pre-CPB CS lactate concentrations were similar between patients who did and did not develop POAF (1.0 [IQR, 0.9-1.4] mmol/L vs 1.1 [IQR, 0.9-1.3] mmol/L, *P* = .66). CS lactate increased significantly from pre-CPB to pre-XC time points (median +1.8 mmol/L [IQR, 0.8-2.5], *P* < .001), with no significant difference in the magnitude of increase between patients with and without POAF (+1.9 mmol/L [IQR, 0.8-2.3] vs +1.7 [IQR, 0.8-2.5], *P* = .97). In contrast, pre-XC concentrations (3.0 [IQR, 1.8-3.6] vs 2.7 [IQR, 2.0-3.8] mmol/L, *P* = .98) were also comparable between groups. After aortic XC removal, there was no change in CS lactate concentrations for patients who developed POAF (−0.3 mmol/L [IQR, −1.0 to 0.5], *P* = .30), whereas a modest decrease was noted among patients who did not develop POAF (−0.7 mmol/L [IQR, −1.4 to −0.1], *P* < .001). However, post-XC CS lactate was significantly greater among patients who developed POAF compared with those who did not (2.4 [IQR, 1.9-3.5] vs 2.0 [IQR, 1.6-2.5] mmol/L, *P* = .02).

A nonlinear relationship between CS lactate concentrations and the incidence of POAF was observed at the post-XC time point (odds ratio, 2.18 mmol/L; 95% CI, 1.21-3.95; *P* = .010). An inflection point at 3 mmol/L was identified and used for subsequent analyses (Figure 2). Patients with post-XC CS lactate >3 mmol/L (n = 13/94, 14%) experienced significantly greater rates of POAF compared with those without elevation (62% vs 20%, *P* = .001). Post-XC CS lactate >3 mmol/L demonstrated high specificity (93%) but modest sensitivity (33%) for POAF prediction, with positive and negative predictive values of 62% and 80%, respectively.

After adjustment for age, number of distal anastomoses, CPB time, XC duration, and earlier lactate measurements, post-XC CS lactate >3 mmol/L remained independently associated with POAF (aOR, 11.7; 95% CI 1.80-76; *P* = .010, Table 2). Increasing age was linked to greater odds of POAF (aOR, 1.14 per year; 95% CI, 1.05-1.23; *P* = .002). CPB time, XC duration, pre-CPB CS lactate, and pre-XC CS lactate were not independently associated with POAF. The model demonstrated excellent discrimination (C-statistic, 0.87; 95% CI, 0.77-0.97).

**Table 2:** Multivariable logistic regression model of predictors of postoperative atrial fibrillation

Variable	aOR	95% CI	*P*
Post-XC CS lactate >3 mmol/L	11.7	1.80-76	.010
Age at operation (per year)	1.14	1.05-1.23	.002
Number of distal anastomoses (per vessel)	1.06	0.40-2.85	.90
Cardiopulmonary bypass time (per min)	1.03	0.99-1.06	.13
Aortic XC time (per min)	0.97	0.93-1.02	.20
Pre-CPB CS lactate (per mmol/L)	0.52	0.07-3.96	.53
Pre-XC CS lactate (per mmol/L)	0.73	0.40-1.35	.32

*aOR*, Adjusted odds ratio; *XC*, crossclamp; *CS*, coronary sinus; *CPB*, cardiopulmonary bypass.

### Factors Associated With Post-XC CS Lactate Concentrations

Patient age and post-XC CS lactate levels were not significantly correlated (r = −0.02, *P* = .82). As shown in Figure 3, there was a positive correlation between the number of distal anastomoses and post-XC CS lactate levels. Specifically, each additional bypass graft was associated with a 0.25 mmol/L increase in lactate concentrations (95% CI, 0.03-0.47; *P* = .028). Furthermore, higher post-XC CS lactate was linked to longer CPB time (r = 0.23, *P* = .024), but not with aortic XC duration (r = 0.16, *P* = .12). Increasing patient BMI was also associated with greater post-XC CS levels (r = 0.33, *P* = .001).

**Figure 3:**
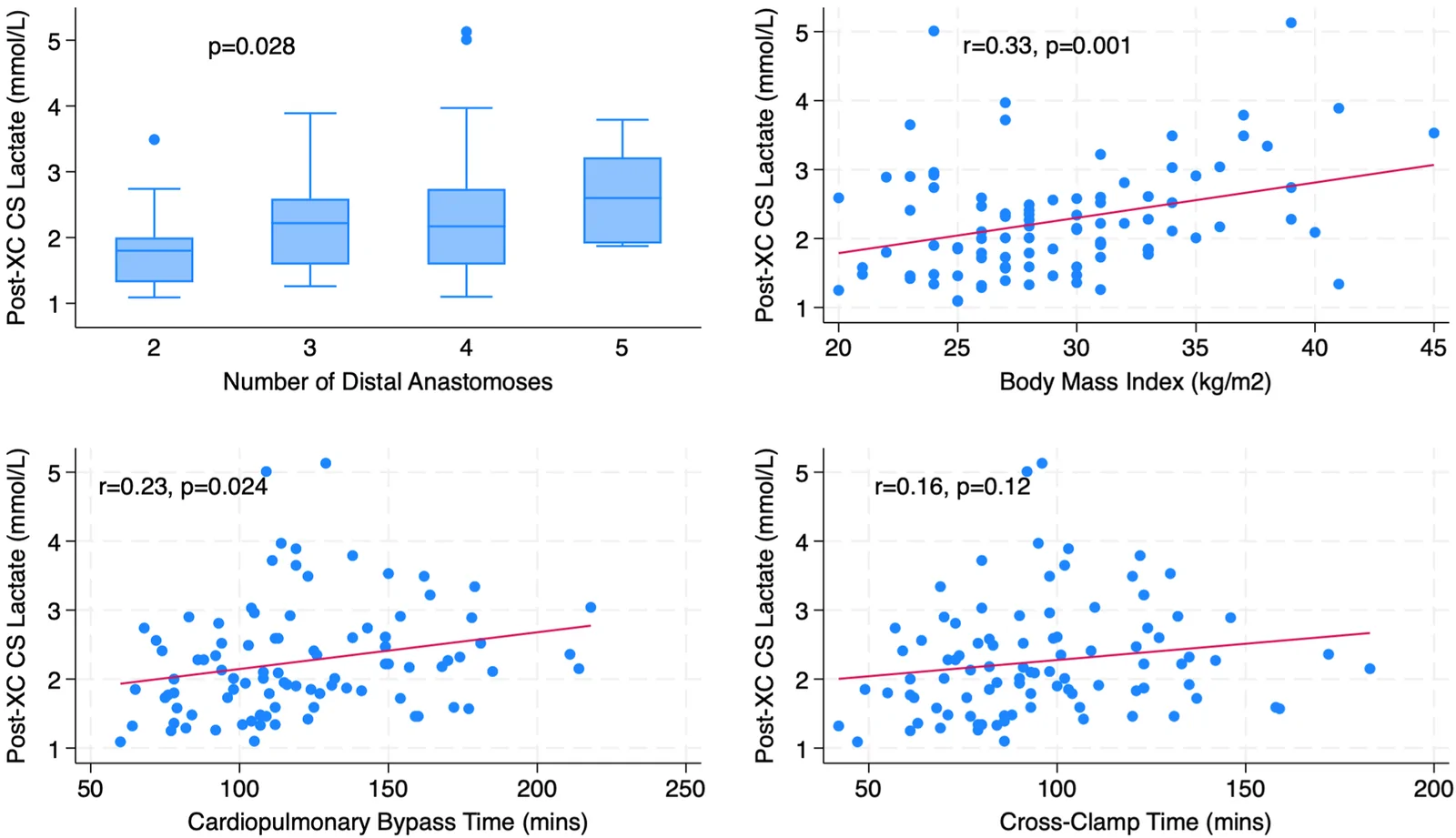
Patient and operative factors correlated with coronary sinus (*CS*) lactate concentrations after aortic crossclamp removal (*post-XC*). *Points* indicate the regression estimate, whereas *bars* represent 95% CIs.

### Sensitivity Analyses

A sensitivity analysis incorporating all patients who developed POAF regardless of treatment with amiodarone was performed. Overall, 37% of patients had electrocardiographic evidence of AF after surgery. Upon unadjusted analysis, increasing post-XC CS lactate was associated with greater odds of POAF (odds ratio, 1.90; 95% CI, 1.09-3.30; *P* = .024). Patients with post-XC CS lactate >3 mmol/L continued to have greater rates of POAF, compared with patients with post-XC CS lactate ≤3 mmol/L (69% vs 32%, *P* = .010). This finding persisted upon multivariable adjustment, with post-XC CS lactate >3 mmol/L associated with a 9.50-fold increase the odds of POAF (95% CI, 1.52-59.6; *P* = .016).

Given the strong correlation between post-XC CS and RA lactate concentrations (r = 0.88, *P* < .001), a secondary analysis evaluated RA lactate >3 mmol/L as a predictor of POAF. Although the RA-based model demonstrated good discrimination, performance was slightly inferior to the CS-based model (C-statistic, 0.82; 95% CI, 0.72-0.92 vs 0.87; 95% CI, 0.77-0.97), supporting the incremental predictive value of CS lactate sampling.

Sensitivity analyses were also conducted to explore the relationship between post-XC CS lactate, age, and POAF. The combination of age and post-XC CS lactate improved discrimination compared with either predictor alone (age only, 0.68; 95% CI, 0.53-0.82; and post-XC CS only, 0.63; 95% CI, 0.53-0.73, both: 0.77, 95% CI, 0.66-0.88; *P* < .001). Predicted probability modeling further demonstrated a graded increase in POAF risk with increasing post-XC CS lactate across all age groups, with older patients exhibiting higher baseline risk (Figure 4).

**Figure 4:**
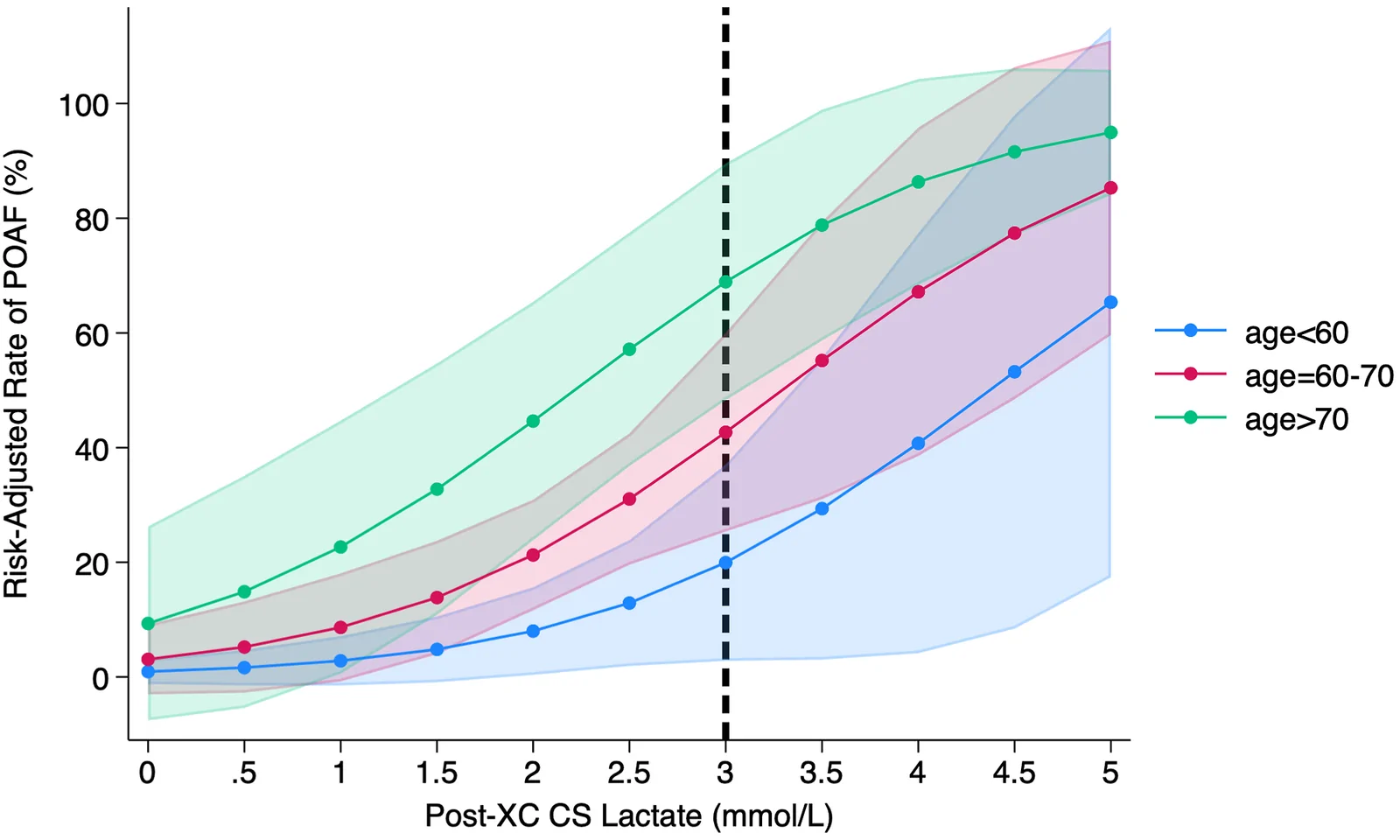
Risk-adjusted predicted probability of postoperative atrial fibrillation (*POAF*) across increasing post-crossclamp (*post-XC*) coronary sinus (*CS*) lactate levels stratified by age.

## Discussion

The present study investigated the association of CS lactate levels with outcomes after isolated CABG. Importantly, we identified a positive, yet nonlinear, relationship between CS lactate concentrations after aortic XC removal and the development of POAF. An inflection point was identified at 3 mmol/L, and patients who exceeded this threshold faced a nearly 12-fold increased risk of POAF, compared with others. Of note, CS lactate concentrations increased substantially for all patients during the ischemic period, but on average, patients who developed POAF were more likely to have sustained elevations in CS lactate despite cardiac reperfusion with XC removal. Several factors were found to be associated with post-XC CS lactate elevation, including greater BMI, increasing number of distal bypassed vessels, and longer CPB duration. Our findings may have significant implications for the perioperative management of patients who undergo cardiac surgery.

Initial studies examining CS lactate concentrations were focused on the diagnosis of coronary artery disease and evaluating the efficacy of cardioplegic solutions.^11^, ^12^, ^13^, ^14^, ^15^ However, there is only a limited body of literature describing the impact of CS lactate levels on cardiac surgical outcomes, especially POAF. In 2001, Rao and colleagues^16^ examined a cohort of 623 patients and found a positive association between post-XC CS lactate and the development of low cardiac output syndrome. A similar prospective study by Onorati and colleagues^17^ further demonstrated that CS lactate levels were linked to altered odds of perioperative myocardial infarction. Within a cohort of 142 patients undergoing noncoronary cardiac operations, Raicea and colleagues^18^ collected serial samples of CS lactate before, during, and after aortic XC. They demonstrated that the incidence of low cardiac output syndrome was significantly greater in patients with hyperlactatemia exceeding 4 mmol/L. Taken together, the existing literature suggests that elevations in CS lactate levels are associated with postoperative myocardial injury with clinically significant implications.^19^

Serial CS sampling revealed a marked increase in lactate concentrations from baseline (pre-CPB) to the pre-reperfusion (pre-XC) time point representing the end of the ischemic period and partial revascularization of the left internal mammary territory. This observation likely reflects the expected transition of cardiomyocytes from aerobic to anaerobic metabolism under cardioplegic arrest.^20^ After complete reperfusion, CS lactate levels remained elevated among patients who developed POAF but decreased for those who did not develop this complication. It is important to note that the increase in CS lactate between the pre-CPB and pre-XC time points was not associated with the onset of POAF, whereas post-XC CS lactate concentrations were significantly correlated with POAF. Taken together, these data allude to the contribution of reperfusion injury rather than ischemic injury as a primary mechanism in the onset of this complication.

Indeed, this study found that CS lactate concentrations >3 mmol/L after aortic XC removal were significantly associated with greater likelihood of POAF. Interestingly, patients who ultimately developed POAF demonstrated persistently elevated CS lactate levels after cardiac reperfusion (post-XC), whereas those who remained free from POAF generally exhibited a decline in lactate concentrations. This study cannot directly comment on the mechanisms underlying the discrepancy between pre-XC and post-XC lactate levels, but our findings imply that some event at the time of reperfusion predisposes patients to AF. Moreover, this event may, in part, play a role in the clearance or continued production of lactate by the myocardium, despite being under aerobic conditions. Previous studies of reperfusion-dependent cardiomyocyte damage have demonstrated that mitochondrial dysfunction, pyruvate dehydrogenase inhibition, and aberrant microcirculation contribute to persistent anaerobic metabolism.^21^, ^22^, ^23^, ^24^, ^25^ Sandler and colleagues^26^ also demonstrated the significance of mitochondrial damage as a consequence of CPB and found higher levels of markers of mitochondrial damage to be linked to an increased risk of POAF. To our knowledge, this work is the first to establish a correlation between CS lactate levels and the incidence of POAF. Future studies are necessary to delineate the molecular mechanisms that underlie our study findings.

Interestingly, overall CPB duration, but not ischemic time, was associated with elevated post-XC CS lactate. We cautiously interpret this result to imply that the myocardial protection strategies used for patients in this study were adequate. In contrast, total bypass time is more complex and may reflect variations in myocardial recovery and the development of vasoplegia. Despite advances in biomaterials engineering, exposure to the CPB circuit is known to incite an inflammatory response, with consequent cytokine release, complement activation, and microvascular dysfunction.^27^, ^28^, ^29^, ^30^ This may, in part, explain the delayed resumption of aerobic metabolism in patients with longer bypass duration, leading to greater post-XC CS lactate values. Although we did not observe a direct correlation between bypass time and the incidence of POAF, a heightened inflammatory state is also known to trigger foci of electrical instability in the atria.^26^^,^^31^

The pathogenesis of POAF is thought to begin with a vulnerable atrial substrate, secondary to age, elevated BMI, valvular dysfunction, and genetic polymorphisms.^32^ Subsequently, intraoperative manipulation of the atria, ischemic and reperfusion injury, as well as systemic inflammatory cascades incited by CPB contribute to the induction and maintenance of AF.^32^, ^33^, ^34^, ^35^ Despite a growing understanding of the etiology of POAF, accurate prognostication in the clinical setting remains elusive. Fields and colleagues^36^ conducted a systematic review of existing risk models and found low discriminatory power (C-statistic, 0.51-0.77) and high risk of bias. Models included in their analysis incorporate validated preoperative risk factors for POAF but do not directly account for the patient- and myocardium-specific impact of CPB. Our study demonstrates that post-XC CS samples can be used to accurately predict the risk of POAF, which can then guide the deployment of targeted mitigation strategies such as amiodarone prophylaxis, beta-adrenergic blockade, or posterior left pericardiotomy.^2^^,^^32^ Importantly, most episodes of POAF occur within postoperative days 2 to 4, and thus, additional information obtained during the intraoperative period would allow for preventative action to be taken in appropriately selected high-risk patients.^3^ Furthermore, for surgeons who do not routinely place a retrograde cardioplegia catheter, this work demonstrated that RA samples can also be used with acceptable ability to predict POAF. The results of our sensitivity analysis suggest that age modifies the absolute risk conferred by post-XC CS lactate elevation, with older patients facing a substantially greater baseline risk of POAF at any given lactate concentration. Consequently, surgeons may adopt age-stratified cutoffs for post-XC CS lactate levels to inform POAF prophylaxis.

### Limitations

Several limitations of this study warrant consideration. First, this analysis was conducted at a single academic medical center and reflects the practice of 2 surgeons. The definition of POAF in this study was determined on the basis of the clinical decision to administer amiodarone, introducing potential for practice-pattern bias. As such, these findings may not be generalizable to other cardiac surgical practices. Second, the modest sample size of 94 patients constrains statistical power, and the findings should be interpreted with appropriate caution. Subgroup analyses could not be performed for this reason. Third, the threshold of 3 mmol/L was derived empirically from restricted cubic spline analysis within this dataset, rather than from a priori clinical rationale. Such data-driven cutoffs carry the risk of overfitting and may not be generalizable in future cohorts. Finally, telemetry surveillance was discontinued at the time of hospital discharge; consequently, late-onset AF occurring after discharge is not captured, and the true incidence of POAF in this cohort may be underestimated.

## Conclusions

In summary, the present study analyzed CS lactate concentrations at varying time points during CABG and demonstrated that elevations >3 mmol/L after cardiac reperfusion were associated with a greater likelihood of POAF. Although our findings represent a promising step forward in the accurate prognostication of AF after cardiac surgery, more studies are necessary to validate our findings in a larger, generalizable cohort. For instance, this work may be expanded with metabolic profiling of the CS serum samples in order to delineate the molecular mechanisms responsible for the link between lactate and AF. Alternatively, more study is necessary to understand the drivers of POAF among patients who did not have elevated post-XC CS lactate concentrations.

### Webcast

You can watch a Webcast of this AATS meeting presentation by going to: https://www.aats.org/resources/coronary-sinus-lactate-at-repe-12224.

**Figure:**
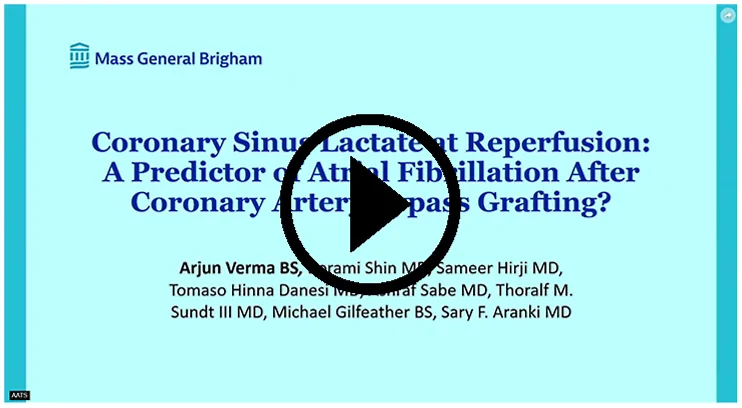

## Conflict of Interest Statement

The authors reported no conflicts of interest.

The *Journal* policy requires editors and reviewers to disclose conflicts of interest and to decline handling or reviewing manuscripts for which they may have a conflict of interest. The editors and reviewers of this article have no conflicts of interest.

## Figures and Tables

**Figure E1:**
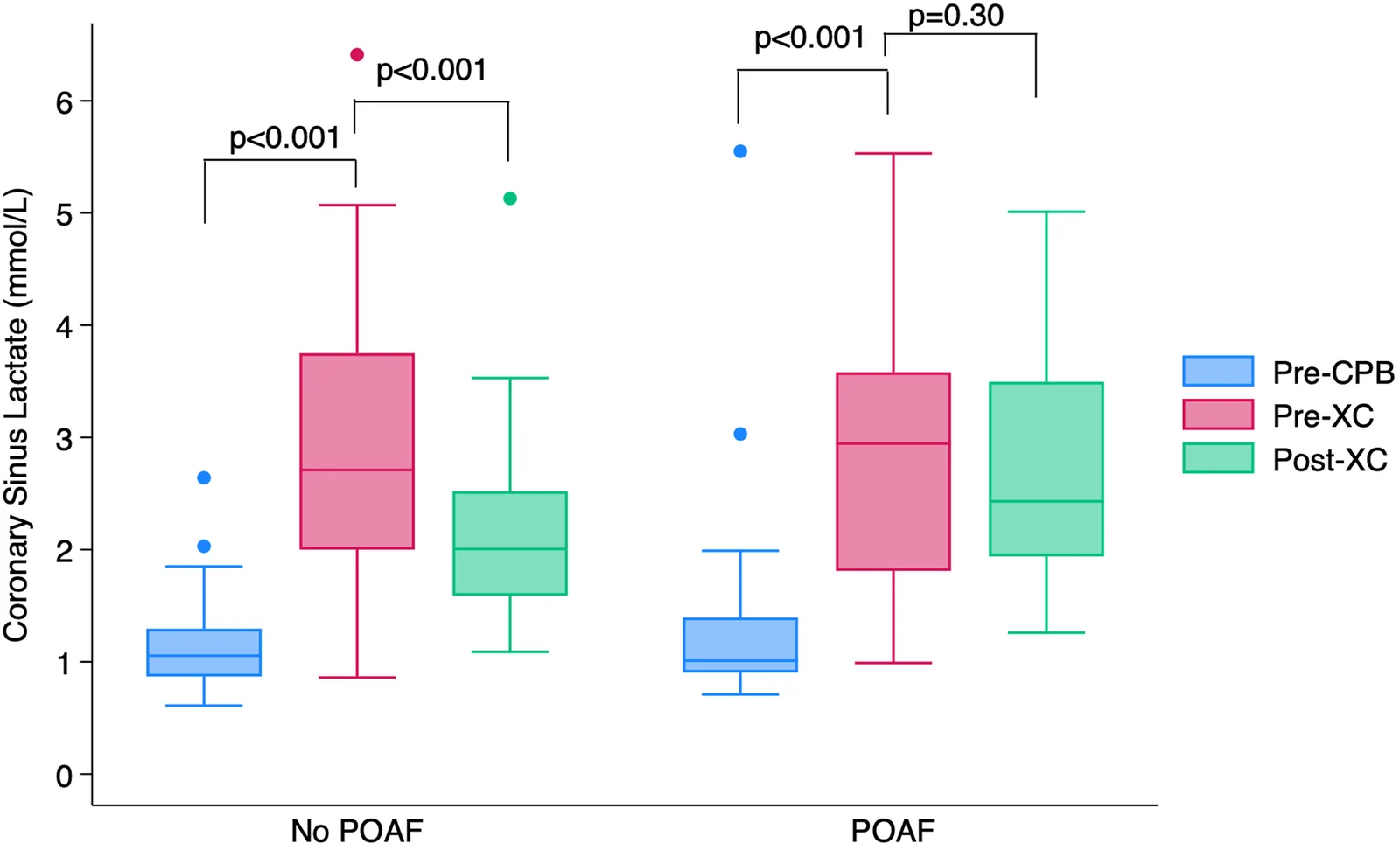
Coronary sinus lactate concentrations before cardiopulmonary bypass (*pre-CPB*), before aortic crossclamp removal (*pre-XC*), and 5 minutes after cardiac reperfusion (*post-XC*), stratified by the occurrence of postoperative atrial fibrillation (*POAF*). *P* values represented paired *t* tests between time points. *Individual dots* represent patient-level data points. The *middle bar* represents 50th percentile, *upper bar* represents the 75th percentile, and *lower bar* represents the 25th percentile. *The upper whisker* corresponds to the 75th percentile plus 1.5 times the interquartile range, whereas the *lower whisker* corresponds to the 25th percentile minus 1.5 times the interquartile range.
